# Reply to the Letter to the Editor by Li et al.: Bioinformatics Analysis in Mice with Diet-Induced Nonalcoholic Steatohepatitis Treated with Astaxanthin and Vitamin E

**DOI:** 10.3390/ijms18050994

**Published:** 2017-05-05

**Authors:** Masuko Kobori, Yumiko Takahashi, Mutsumi Sakurai, Yinhua Ni, Guanliang Chen, Mayumi Nagashimada, Shuichi Kaneko, Tsuguhito Ota

**Affiliations:** 1 Food Research Institute, National Agriculture and Food Resrach Organization, Tsukuba, Ibaraki 305-8642, Japan; yumitala@affrc.go.jp (Y.T.); imustum@affrc.go.jp (M.S.); 2 Department of Cell Metabolism and Nutrition, Brain/Liver Interface Medicine Research Center, Kanazawa University, Kanazawa, Ishikawa 920-8640, Japan; shali0145@gmail.com (Y.N.); guanliangc@gmail.com (G.C.); nakanaga@staff.kanazawa-u.ac.jp (M.N.); skaneko@m-kanazawa.jp (S.K.)

## Reply:

Thank you for the valuable comments. The purpose of our study is not to prove the significant difference between the groups. We have already reported that astaxanthin (AX) alleviated high-cholesterol, high-cholate, and high-fat (CL) diet-induced NASH and it was more effective than vitamin E in preventing and treating NASH in mice [1]. In this paper, we showed possible molecular action of AX on CL diet-induced NASH. This is important for the safe use of AX as a new therapy. On this point, we do not think that we need to use “a fewer false positive method”, which may lead to a higher false negative rate. According to the statement released by the American Statistical Association, scientific conclusions should not be based only on whether a *p*-value passes a specific threshold [2]. In addition, we performed qPCR analysis and showed that astaxanthin significantly altered the mRNA expression associated with EIF2 signaling, mitochondrial dysfunction, PPARα and PPARγ. We think that further study is necessary to elucidate the molecular mechanism of AX and to establish a new therapy for patients with NASH.
